# Mexican Oregano Infusion and Carvacrol Modify Cytokine Concentration, Induce Apoptosis, and Regulate Antioxidant Enzyme Expression and Function in Breast Cancer Cell Lines: Implications for Bioactive Molecule-Based Therapeutics

**DOI:** 10.3390/antiox15101345

**Published:** 2026-10-17

**Authors:** Brian Enrique Rojo-Ruvalcaba, Montserrat Maldonado-González, Bertha Ruiz-Madrigal, Erika Martínez-López, Rocío García-Becerra, Pedro Ernesto Sánchez-Hernández, Juan Florencio Gómez-Leyva, Teresa Arcelia García-Cobián, María Guadalupe Ramírez-Dueñas, Andrea Carolina Machado-Sulbaran, Héctor Flores-Martínez, Itzel Viridiana Reyes-Pérez, Eduardo Huerta-García, Cristina Estefanía Rizo-Estrada, Alejandro Ulises Centeno-Torres, Trinidad García-Iglesias

**Affiliations:** 1 Departamento de Clínicas de la Reproducción Humana, Crecimiento y Desarrollo Infantil, Instituto de Cáncer en la Infancia y la Adolescencia (INICIA), Centro Universitario de Ciencias de la Salud (CUCS), Universidad de Guadalajara, Guadalajara 44340, Jalisco, Mexico; brian.rojo0999@alumnos.udg.mx (B.E.R.-R.); pedro.shernandez@academicos.udg.mx (P.E.S.-H.); andrea.machado5223@academicos.udg.mx (A.C.M.-S.); eduardo.huerta0813@alumnos.udg.mx (E.H.-G.); cristina.rizo5650@alumnos.udg.mx (C.E.R.-E.); alejandro.centeno4231@alumnos.udg.mx (A.U.C.-T.); 2 Departamento de Microbiología y Patología, Centro de Investigación en Enfermedades Infectocontagiosas (CIEIC), Centro Universitario de Ciencias de la Salud (CUCS), Universidad de Guadalajara, Guadalajara 44340, Jalisco, Mexico; montserrat.maldonado@academicos.udg.mx (M.M.-G.); bertha.ruiz@academicos.udg.mx (B.R.-M.); 3 Instituto de Nutrigenética y Nutrigenómica Traslacional (INNUGET), Centro Universitario de Ciencias de la Salud (CUCS), Universidad de Guadalajara, Guadalajara 44340, Jalisco, Mexico; erika.martinez@academicos.udg.mx; 4 Departamento de Biología Molecular y Biotecnología, Instituto de Investigaciones Biomédicas, Universidad Nacional Autónoma de México, Ciudad de México 04510, Mexico; rocio.garciab@iibiomedicas.unam.mx; 5 Programa de Investigación de Cáncer de Mama, Instituto de Investigaciones Biomédicas, Universidad Nacional Autónoma de México, Ciudad de México 04510, Mexico; 6 Departamento de Fisiología, Centro Universitario de Ciencias de la Salud (CUCS), Universidad de Guadalajara, Guadalajara 44340, Jalisco, Mexico; maria.rduenas@academicos.udg.mx; 7 Cuerpo Académico UDG-CA-1135 “Biomarcadores Inmunogenéticos y Factores Farmacológicos en Enfermedades Crónico-Degenerativas”, Centro Universitario de Ciencias de la Salud (CUCS), Universidad de Guadalajara, Guadalajara 44340, Jalisco, Mexico; itzel.reyes@academicos.udg.mx; 8 DEPI, Instituto Tecnológico de Tlajomulco, Tecnológico Nacional de México, Tlajomulco de Zúñiga 45640, Jalisco, Mexico; juan.gl@tlajomulco.tecnm.mx (J.F.G.-L.); hector.fm@tlajomulco.tecnm.mx (H.F.-M.); 9 Instituto de Terapéutica Experimental y Clinica (INTEC), Departamento de Fisiología, Centro Universitario de Ciencias de la Salud (CUCS), Universidad de Guadalajara, Guadalajara 44340, Jalisco, Mexico; arcelia.garcia@academicos.udg.mx; 10 Instituto de Investigación en Reumatología y del Sistema Músculo Esquelético (IIRSME), Centro Universitario de Ciencias de la Salud (CUCS), Universidad de Guadalajara, Guadalajara 44340, Jalisco, Mexico; 11 Departamento de Salud Pública, Instituto Regional de Investigación en Salud Pública, Centro Universitario de Ciencias de la Salud (CUCS), Universidad de Guadalajara, Guadalajara 44340, Jalisco, Mexico

**Keywords:** anticancer agents, alternative treatments, breast cancer, redox balance, cytokine secretion, complementary treatments

## Abstract

**Background/Objectives:** Breast cancer (BC), particularly aggressive subtypes such as triple-negative TNBC, remains a significant clinical challenge due to treatment resistance and therapeutic toxicity. This study evaluated the antitumor potential, antioxidant activity, and anti-inflammatory effect of Mexican Oregano (*Lippia graveolens*) infusion (MoI) and its bioactive constituent, carvacrol (Cv), in diverse BC cell lines (MCF-7, HCC-1954, and MDA-MB-231). Methods: Cellular responses were assessed by quantifying apoptosis, profiling cytokines (IL-4, IL-6, IL-10), and analyzing the gene expression of antioxidant enzymes (*CAT, SOD2, GPX1*) and their enzymatic activities. Results: Cv exhibited potent, subtype-independent pro-apoptotic activity, while MoI demonstrated significant efficacy with attenuated cytotoxicity in non-tumoral HaCaT cells. Both treatments mediated complex modification of cytokine signaling, characterized by reduction in the extracellular concentration of tumor-promoting IL-4, IL-6, and IL-10, an effect most pronounced in the highly aggressive MDA-MB-231 cell line. Transcriptional and functional analyses further revealed that these agents alter the expression and activity of antioxidant enzymes, thereby promoting pro-oxidant environments conducive to cancer cell death. Conclusions: These findings indicate that MoI and Cv exert antitumor effects through coordinated pathways of apoptosis induction, extracellular cytokine levels and redox disruption. Consequently, MoI emerges as a promising candidate for complementary therapeutic strategies in BC management, particularly for aggressive subtypes, owing to its impact on inflammatory networks and redox vulnerabilities. At the same time, Cv serves as a compelling lead compound for targeted oncological research.

## 1. Introduction

Breast Cancer (BC) is one of the leading causes of cancer-related morbidity and mortality in women worldwide, making it a major public health issue. Despite advances in conventional therapeutic strategies such as surgery, radiation therapy, chemotherapy, endocrine therapy, and targeted therapies, challenges persist regarding treatment resistance, side effects, and poor prognosis in aggressive subtypes, such as TNBC. This subtype lacks effective therapeutic targets and is typically managed with chemotherapy, which is known for its aggressiveness and lack of specificity. This highlights the need to develop new therapeutic strategies. In this regard, the study of plant-derived extracts and compounds has emerged as a promising area for research and development of alternatives for the management of neoplastic disease [1,2,3].

In phytomedicine, *Lippia graveolens Kunth*, commonly known as Mexican oregano, has been widely used in traditional medicine for its antimicrobial, anti-inflammatory, and antioxidant properties. One of the compounds present in the highest amounts in extracts of various oregano species is carvacrol (Cv). This monoterpenoid phenol has attracted attention for its cytotoxic and proapoptotic properties in various cancer cell lines, including BC, acting through multiple molecular pathways. For example, it has been shown to induce apoptosis via the intrinsic pathway by inhibiting the expression of anti-apoptotic proteins such as Bcl-2, altering mitochondrial membrane potential, and promoting the release of cytochrome C [4]. Furthermore, it has been reported to modulate tyrosine kinase receptor (TKR) signaling, in part by inhibiting the TRPM7 cation channel, which is involved in the homeostasis of divalent cations such as magnesium, calcium, and zinc, and in the activation of oncogenic pathways such as PI3K/AKT, MAPK, and JAK/STAT. Likewise, Cv induces oxidative stress by increasing the production of reactive oxygen species (ROS), thereby disrupting redox homeostasis and compromising cell viability. However, the mechanism of action of MoI remains unknown, as does relevant information regarding Cv, such as modulation of the tumor microenvironment via changes in levels of key cytokines involved in disease progression and dissemination, or the alterations that lead to increased ROS levels [3,5,6,7,8,9,10].

Therefore, the objective of this study was to evaluate how MoI or Cv treatment modifies redox balance and cytokine secretion and how these alterations may lead to apoptosis in breast cancer cell lines of distinct molecular subtypes (MCF-7, HCC-1954, and MDA-MB-231).

## 2. Materials and Methods

### 2.1. Chemical and Reagents

Cv ≥98% purity (Cat. No. 499-75-2) and dimethyl sulfoxide (DMSO, Cat. No.D8418) were purchased from Sigma-Aldrich (St Louis, MO, USA). Dulbecco’s Modified Eagle Medium (DMEM, Cat. No. 11966025), Roswell Park Memorial Institute (RPMI-1640, Cat. No. 11875119), phosphate-buffered saline (PBS, Cat. No. 10010023), fetal bovine serum (FBS, Cat. No. 16140071), penicillin/streptomycin (Cat. No. 15140122), and trypsin (Cat. No. 25300054) were purchased from Thermo Fisher ScientificTM (Waltham, MA, USA). All solvents and chemicals were of analytical grade.

### 2.2. Cell Lines and Culture Conditions

Cell lines were purchased from American Type Culture Collection (ATCC; Manassas, VA, USA). Immortalized human keratinocyte HaCaT cells and TNBC MDA-MB-231 cells were maintained in RPMI-1640; luminal A MCF-7 and HER2 HCC-1954 BC cell lines were maintained in DMEM; both media were supplemented with 10% FBS and 1% penicillin/streptomycin. The cell cultures were kept in incubation under physiological conditions (37 °C, 95% humidity, and 5% CO2 saturation) until approximately 80% confluence was observed, at which point the cells were harvested by trypsinization.

### 2.3. Plant Material

Mexican oregano (*Lippia graveolens Kunth*) leaves used in this study were obtained from the region of Totatiche, Jalisco, Mexico. A voucher specimen (No. SIST-TRA-20250506) was deposited at the Instituto de Botánica de la Universidad de Guadalajara (IBUG) of Jalisco, Mexico (supply plant description information).

### 2.4. Aqueous Extraction of Mexican Oregano (Lippia graveolens Kunth)

A volume of 3 mL distilled water was heated to boiling. After removing from the heat, 69 mg of ground oregano leaves were added. The mixture was covered with aluminum foil to prevent loss of volatile and left at room temperature for 10 min. It was then filtered through a 0.22 µm syringe filter (the concentration of carvacrol was evaluated by HPLC as 0.607 mg/dL, thymol 51.48 mg/dL, 126 µg/mL galagnina and 1.001 µg/mL of pinocembrina in the Mol infusion; previously reported data) [3].

The previously reported IC_50_ for each cell line was used for the assays in this study (HaCaT: 0.18 mg/mL, MCF-7: 0.08 mg/mL, HCC-1954: 0.15 mg/mL, and MDA-MB-231: 0.17 mg/mL, with a treatment time of 48 h) [3].

### 2.5. Preparation of Cv Solution

A volume of 1.6 µL of Cv was mixed with 10.6 µL of DMSO and 1988 µL of culture medium to obtain a 1000µM solution.

The previously reported IC_50_ for each cell line was used for the assays in this study (HaCaT: 110 µM, MCF-7: 211 µM, HCC-1954: 123 µM, and MDA-MB-231: 121 µM, with a treatment time of 24 h) [3].

### 2.6. Apoptosis Assessment

Apoptosis was quantified by measuring cytoplasmic histone-associated DNA fragments (mono- and oligonucleosomes) using the Cell Death Detection ELISA kit (Roche, Cat. No. 11544675001) according to the manufacturer’s instructions. Briefly, cell cultures were harvested and centrifuged at 300× *g* for 10 min to obtain cell pellets and supernatants. The cell pellets were resuspended and incubated in the provided lysis buffer for 30 min at room temperature to release cytoplasmic fractions, leaving the nuclei intact. Subsequently, the lysate was centrifuged at 20,000× *g* for 10 min, and 400 µL of the supernatant (=cytoplasmic fraction) was carefully removed for further analysis. Afterward, 100 µL of coating solution was pipetted into each well of the microplate modules, tightly covered with self-adhesive foil, and incubated for 1 h. After thorough removal of the coating solution, 200 µL of incubation buffer was added to each well for 30 min to block nonspecific binding sites. Wells were then rinsed three times with 300 µL of washing solution. Subsequently, 100 µL of the sample solution or negative control was added to the wells. For immunoassay background determination, 100 µL of incubation buffer was added to each well. The plates were covered and incubated for 90 min. Following another three-wash cycle, 100 µL of conjugate solution was added to each well (except for the blank position) and incubated for 90 min. After a final washing step, 100 µL of substrate solution was pipetted into each well. The microplate was incubated on a plate shaker for approximately 10–20 min until sufficient color development was observed. After gently tapping the side of the microplate to homogenize the contents, the optical density was measured at 405 nm against a substrate solution blank (reference wavelength 490 nm) using a microplate reader (BioTek-800TS, Winooski, VT, USA).

### 2.7. Determination of the Cytokines IL-4, IL-6, and IL-10 in the Cell Supernatant

Supernatants from cell cultures were harvested (5 × 10^5^ cells in a 6-well culture plate under the corresponding treatment), centrifuged at 300× *g* for 10 min at 4 °C to remove cellular debris, and stored in aliquots at −80 °C until analysis. The extracellular concentrations of IL-4, IL-6, and IL-10 were quantified using commercial sandwich ELISA kits (ELISA MAX™ Standard Sets; BioLegend, San Diego, CA, USA; Cat. Nos. 430301, 430501, and 430601, respectively) according to the manufacturer’s instructions.

Briefly, 96-well high-binding microplates were coated with the respective capture antibodies overnight at 4 °C. Nonspecific binding sites were blocked using the assay buffer provided in the kit for 1 h at room temperature. Following the blocking step, cell culture supernatants and a series of twofold dilutions of the protein standards were added to the wells and incubated for 2 h at room temperature under shaking. Plates were washed extensively four times between steps with PBS containing 0.05% Tween-20.

Subsequently, the specific biotinylated detection antibodies were added and incubated for 1 h, followed by a 30-min incubation with Avidin-HRP conjugate. The colorimetric reaction was developed by adding tetramethylbenzidine (TMB) substrate solution in the dark and stopped after 15–20 min by the addition of stop solution. The optical density of each well was immediately measured at 450 nm, with a wavelength correction set at 570 nm, using a microplate spectrophotometer (BioTek-800TS, Winooski, VT, USA). Cytokine concentrations were calculated from the standard curves utilizing a four-parameter logistic regression curve-fit.

### 2.8. RNA Extraction

Total RNA was extracted from each study group using TRIzol^®^ (Invitrogen, Cat. No. 10296010). RNA quantity and purity were estimated using a Nanophotometer P-Class (Implen Inc., München, Germany). The integrity of samples was tested by 1% agarose gel electrophoresis. One μg of total RNA was reverse-transcribed in a total volume of 20 μL using the High-Capacity cDNA Reverse Transcription Kit (Applied Biosystems, Cat. No. 4368814) and RNaseOUT Ribonuclease Inhibitor (Invitrogen, Cat. No. 10777019), following the manufacturer’s instructions. The resulting cDNA samples were stored at −20 °C until further analysis.

### 2.9. Real-Time qPCR

RT-qPCR reactions were performed using 5 μL FastStart Essential DNA Probes Master Mix (Roche, Cat. No. 06402682001), 2.5 μL H2O, 0.5 μL hydrolysis probes (TaqMan^®^, Thermo Fisher Scientific), and 2 μL of cDNA as a PCR template for a total reaction volume of 10 μL. TaqMan^®^ probes for each gene of interest were as follows: *GPX1* (ID: Hs00829989_gH), *SOD2* (ID: Hs00167309_m1), and *CAT* (ID: Hs00156308_m1). To identify and validate the reference genes, we tested: *ACTB* (ID: Hs99999903_m1), *GAPDH* (ID: Hs99999905_m1), and *POLR2A* (ID: Hs00172187_m1). All RT-qPCR assays were performed in a LightCycler^®^ 96 Real-Time PCR System (Roche Diagnostics, Mannheim, Germany) with standard thermal cycling conditions.

To identify and validate the reference genes, serial dilutions of the cDNA were used to generate standard curves. These curves were employed to determine the amplification efficiency (E) and linearity of each assay in each cell line. Amplification efficiency was calculated from the slope of the standard curve using the equation: E = 10^(−1/slope). The coefficient of determination (R^2^) was also calculated to assess the linear relationship between the cDNA input and the quantification cycle values for each assay.

Each run included a no-template control, consisting of sterile distilled water instead of template cDNA. The assays were reproducible when conducted in triplicate, yielding comparable results. Finally, interest gene expression was normalized to *POLR2A* and to the expression of the untreated cell line. Relative gene expression was estimated according to the 2−ΔΔCt method.

### 2.10. Activity of Glutathione Peroxidase

The enzymatic activity was quantified using a glutathione peroxidase assay kit (Cayman Chemical, Cat. No. 703102) according to the manufacturer’s instructions and normalized to a total of 3 × 10^5^ cells at the time of sampling. Briefly, enzymatic activity was assessed indirectly through a coupled reaction with glutathione reductase. Glutathione peroxidase reduces cumene hydroperoxide while oxidizing reduced glutathione (GSH) to glutathione disulfide (GSSG), which is subsequently recycled back to GSH by GR with a stoichiometric consumption of NADPH to *NADP*^+^.

In a 96-well plate, 100 µL of Assay Buffer (50 mM Tris-HCl, pH 7.6, 5 mM EDTA) was combined with 50 µL of co-substrate mixture (NADPH, GSH, and GR) and 20 µL of sample or sample buffer. The reaction was triggered by adding 20 µL of cumene hydroperoxide. Absorbance at 340 nm (BioTek-Synergy HTX, Winooski, VE, USA) was monitored kinetically every minute for 5 min at 25 °C. The linear rate of NADPH disappearance (*ΔA*_340_/min) was used to calculate activity using the NADPH extinction coefficient (*ϵ*_340_ = 6.22 mM^−1^cm^−1^) adjusted for path length. One unit of glutathione peroxidase was defined as the amount of enzyme that oxidizes 1.0 nmol of NADPH to *NADP*^+^ per minute at 25 °C.

### 2.11. Activity of Catalase

The enzymatic activity was quantified using a catalase (CAT) assay kit (Cayman Chemical, Cat. No. 707002) according to the manufacturer’s instructions and normalized to a total of 3 × 10^5^ cells at the time of sampling. Briefly, CAT peroxidatic activity was measured based on the enzyme-mediated oxidation of methanol to formaldehyde in the presence of hydrogen peroxide (*H*_2_*O*_2_). Reactions were prepared in a 96-well plate by combining 100 µL of Assay Buffer (100 mM potassium phosphate, pH 7.0), 30 µL of methanol, and 20 µL of diluted sample, positive control, or formaldehyde standards (0–75 µM). The reaction was initiated with 20 µL of *H*_2_*O*_2_ and incubated for 20 min at room temperature under continuous shaking. The enzymatic reaction was arrested with 30 µL of 10 M KOH, followed by the addition of 30 µL of Purpald chromogen (4-amino-3-hydrazino-5-mercapto-1,2,4-triazole) and incubation for 10 min at room temperature. The formaldehyde–Purpald adduct was subsequently oxidized to a purple product by adding 10 µL of potassium periodate (*KIO*_4_). Absorbance was read at 540 nm (BioTek-Synergy HTX, Winooski, VE, USA). One unit of CAT activity was defined as the amount of enzyme producing 1.0 nmol of formaldehyde per minute at 25 °C.

### 2.12. Activity of Superoxide Dismutase 2

The enzymatic activity was quantified using a commercial superoxide dismutase 2 (SOD2) assay kit (Cayman Chemical, Cat. No. 706002) according to the manufacturer’s instructions and normalized to a total of 3 × 10^5^ cells at the time of sampling. Briefly, to selectively quantify the manganese-dependent mitochondrial isoform (SOD2) and eliminate interference from cytosolic and extracellular Cu/Zn-SODs, subcellular fractionation was carried out. Samples were homogenized in ice-cold isolation buffer (20 mM HEPES, pH 7.4, 210 mM mannitol, 70 mM sucrose, and 1 mM EDTA) and centrifuged at 1000× *g* for 10 min at 4 °C to remove nuclei and unbroken cellular debris. The supernatant was subsequently centrifuged at 10,000× *g* for 15 min at 4 °C to pellet the mitochondrial fraction. Pellets were resuspended in 50 mM Tris-HCl (pH 8.0) and sonicated on ice to lyse mitochondrial membranes and release SOD2. Alternatively, selective inhibition of Cu/Zn-dependent isoforms was performed by pre-incubating total lysates with 1–3 mM potassium cyanide (KCN) for 15–20 min prior to the assay.

For the assay, 10 µL of sample or standard was mixed with 200 µL of tetrazolium salt radical detector diluted in Assay Buffer (50 mM Tris-HCl, pH 8.0, 0.1 mM DTPA, 0.1 mM hypoxanthine) in a 96-well plate. Enzymatic reactions were initiated by adding 20 µL of xanthine oxidase to generate superoxide anion radicals ($O_{2}^{∙-}$). Following incubation for 20 min at room temperature on an orbital shaker, optical density was measured at 450 nm (BioTek-Synergy HTX, Winooski, VE, USA). Activity was interpolated from a standard curve generated with bovine erythrocyte SOD (0–0.25 U/mL). One unit (U) of SOD activity was defined as the amount of enzyme needed to cause 50% dismutation of the superoxide radical.

### 2.13. Statistical Analysis

Assays were conducted in triplicate at different times to ensure reproducibility, yielding comparable results. Data distribution was assessed for normality using the Shapiro–Wilk test. For normally distributed data, results were expressed as means ± standard deviation (SD), and statistical significance was determined using Student’s t-test or one-way ANOVA followed by Dunnett’s post-hoc test. For non-parametric data, equivalent non-parametric tests were applied. Correlations between variables were assessed using Pearson’s correlation coefficient. All statistical analyses were performed using R (version 4.6.0), and a *p*-value < 0.05 was considered statistically significant.

## 3. Results

### 3.1. Apoptosis Induction

Figure 1 shows the relative apoptosis levels across all cell lines following treatment with MoI, Cv, or etoposide, compared to the untreated control. In the non-tumoral HaCaT cell line (Figure 1A), MoI induced a marginal increase in apoptosis (1.21-fold relative to the control). In contrast, Cv elicited a markedly stronger effect, increasing apoptotic activity nearly four-fold. A similar trend was observed across the BC cell lines MCF-7 (Figure 1B), HCC-1954 (Figure 1C), and MDA-MB-231 (Figure 1D); MoI mediated modest increases ranging from 1.32- to 1.40-fold, while Cv stimulated a substantial 3.5- to 3.8-fold upregulation. As expected, the positive control, etoposide, induced the highest apoptotic response across all evaluated cell lines, ranging from 5.3- to 6.1-fold above basal levels.

Notably, the pro-apoptotic response triggered by Cv was consistent among the three BC subtypes, including the highly aggressive TNBC MDA-MB-231 cells.

### 3.2. Cytokine Production

Basal IL-4 production differed among cell lines and was differentially modulated by the treatments (Figure 2A). In HaCaT and HCC-1954 cells, MoI significantly reduced IL-4 concentration, whereas Cv increased IL-4 levels in HaCaT cells and maintained values close to baseline in HCC-1954 cells. In MCF-7 cells, both treatments produced moderate increases in IL-4 compared with untreated controls.

The most pronounced effect was observed in MDA-MB-231 cells, where Cv drastically reduced IL-4 to approximately 20% of basal levels, while MoI produced a modest increase.

Treatment-associated changes in IL-6 production were highly cell type-dependent (Figure 2B). In HaCaT and MCF-7 cells, MoI and Cv induced only modest changes in IL-6; however, these differences were statistically significant. In contrast, Cv markedly increased IL-6 concentration in HCC-1954 cells, resulting in levels nearly fourfold higher than those of the untreated control.

MDA-MB-231 cells exhibited an exceptionally high basal IL-6 concentration, and IL-6 levels following MoI treatment exceeded the assay’s upper limit of detection (>500 pg/mL). Although Cv and etoposide significantly reduced IL-6 concentration in this cell line, cytokine levels remained substantially higher than those observed in the other cellular models.

Distinct patterns of IL-10 regulation were observed among the cell lines evaluated (Figure 2C). In HaCaT cells, MoI significantly increased IL-10 production, whereas Cv induced only minimal changes. In contrast, IL-10 levels remained largely unchanged in MCF-7 and HCC-1954 cells regardless of treatment.

MDA-MB-231 cells exhibited the highest basal IL-10 concentrations. Both MoI and Cv significantly reduced IL-10 secretion, with MoI exerting the strongest inhibitory effect. Etoposide further decreased IL-10 levels.

### 3.3. Reference Gene Validation for RT-qPCR Assay

The standard curves generated for the target genes demonstrated excellent amplification efficiencies of 1.94, 1.87, and 1.99 for *CAT*, *SOD2*, and *GPX1*, respectively, with excellent linearity (R^2^ = 1.00) for all assays. Among the candidate reference genes, *POLR2A* consistently exhibited amplification characteristics most comparable to those of the target genes, with amplification efficiencies of 1.98, 1.85 (R^2^ = 0.99), and 1.98 (R^2^ = 1.00) for *CAT*, *SOD2*, and *GPX1*, respectively. In contrast, *ACTB* and *GAPDH* showed larger deviations in amplification efficiency across all assays. These results indicate that all RT-qPCR assays fulfilled the recommended criteria for amplification performance and support *POLR2A* as the most suitable reference gene for the relative quantification of *CAT*, *SOD2*, and *GPX1* expression under the experimental conditions evaluated.

### 3.4. Gene Expression of Antioxidant Enzymes

Gene expression analysis revealed that MoI and Cv differentially modulated antioxidant defense genes in a cell line-dependent manner, exerting distinct effects on the expression of *CAT*, *GPX1*, and *SOD2* (Figure 3). Expression levels were calculated relative to the corresponding untreated control group for each gene.

In the HaCaT cell line (Figure 3A), MoI significantly downregulated *CAT* and *GPX1* expression by 44.5% and 56.0%, respectively, while markedly upregulating *SOD2* expression by 61.9%. Similarly, Cv increased *SOD2* expression by 35.2%, although its inhibitory effect on *CAT* was less pronounced (14.4%), and it slightly increased *GPX1* expression (16.1%).

In the Luminal A cell line, both treatments significantly reduced *CAT* expression, with decreases of 59.9% and 52.2% following MoI and Cv treatment, respectively. However, MoI increased *GPX1* expression by 26.6%, whereas Cv strongly induced *SOD2* expression (241.1%) while simultaneously suppressing *GPX1* expression by 55.9% (Figure 3B). This contrasting response pattern suggests the activation of distinct antioxidant regulatory pathways by the infusion and its major bioactive constituent.

In HCC-1954 cells (Figure 3C), MoI increased *CAT* and *SOD2* expression by 20.6% and 45.4%, respectively, while reducing *GPX1* expression by 43.0%. In contrast, Cv decreased the expression of all three antioxidant genes, reducing *CAT*, *SOD2*, and *GPX1* expression by 32.9%, 24.8%, and 72.2%, respectively, indicating a broad impact on antioxidant defense mechanisms.

The most pronounced effects were observed in MDA-MB-231 cells (TNBC subtype, Figure 3D). MoI significantly downregulated *CAT*, *SOD2*, and *GPX1* expressions by 57.8%, 76.2%, and 38.0%, respectively. Cv also reduced the expression of these genes by 17.6%, 28.8%, and 17.6%, respectively, although the magnitude of the effect was less pronounced.

### 3.5. Antioxidant Enzyme Activity

Functional analysis of glutathione peroxidase activity partially mirrored the transcriptional findings (Figure 4A). In HaCaT cells, glutathione peroxidase activity increased moderately but significantly following treatment with either MoI or Cv (14.44% and 33.19%, respectively). In contrast, MCF-7 cells exhibited a marked reduction in glutathione peroxidase activity after Cv exposure (72.03%, ** *p* < 0.001), whereas MoI produced a moderate increase. HCC-1954 cells showed a significant increase in glutathione peroxidase activity following MoI treatment (82.7%), while Cv slightly reduced (11.5%) enzyme activity (EA). In the MDA-MB-231 cell line, contrary results were observed: while MoI decreased its activity (18.64%), the Cv treatment slightly increased it (2.97%).

Unlike gene expression data, catalase activity remained remarkably stable across all cell lines and treatment conditions (Figure 4B). Enzyme activity consistently ranged from 0.13 to 0.14 nmol/min/mL, indicating that transcriptional alterations in CAT did not translate into substantial changes in catalytic activity over the evaluated experimental period.

Overall, SOD2 activity exhibited only moderate treatment-induced changes across the cell lines analyzed (Figure 4C). In HaCaT cells, exposure to either MoI or Cv resulted in small but statistically significant decreases in enzymatic activity. In MCF-7 cells, SOD2 activity remained largely unchanged, indicating a limited response to both treatments. Conversely, HCC-1954 cells showed a significant increase in SOD2 activity following MoI treatment (18.80%, *p* < 0.05), in agreement with the observed upregulation of SOD2 transcript levels. In contrast, Cv treatment did not significantly alter enzyme activity relative to basal conditions. In MDA-MB-231 cells, MoI induced a slight reduction in SOD2 activity, whereas Cv had a non-significant effect.

### 3.6. Correlation Heatmap and Hierarchical Clustering

Hierarchical clustering heatmaps were generated using the ClustVis web server. Data were pre-processed using row centering and unit variance scaling. Both rows and columns were clustered using Pearson correlation distance as the distance metric and average linkage. This analysis revealed distinct, cell line-specific immunomodulatory and enzymatic profiles across the four evaluated cell lines (Figure 5). In MDA-MB-231 cells, Cv treatment segregated into an independent clade distinct from control and Mol samples, characterized by lower levels of pro-inflammatory IL-6 and elevated functional enzymatic capacity of SOD2 and GPX1 (SOD2 EA and GPX1 EA). In HaCaT and HCC-1954 cells, Mol treatment formed a unique cluster characterized by lower IL-4 levels relative to control and Cv conditions, accompanied by marked increases in SOD2 and GPX1 enzymatic activities and moderate elevations in IL-10. In MCF-7 cells, both Cv and Mol treatments exhibited high levels of IL-4 alongside decreased GPX1 enzymatic activity compared to baseline control values. Across all four experimental models, mRNA transcript levels of antioxidant enzymes (SOD2, CAT, and GPX1) remained low or near baseline.

## 4. Discussion

The anticancer activity of Cv and oregano-derived extracts, including *Lippia graveolens Kunth*, has been extensively documented in several cancer models, providing substantial evidence of their cytotoxic potential against BC cells [3,5,7,8,9,10]. Building upon this evidence, the present study aimed to characterize the mechanisms underlying these effects by evaluating the induction of apoptosis, concentration of extracellular cytokines, and alterations in antioxidant defense systems. Collectively, our findings indicate that both the MoI and Cv exert antitumor effects through interconnected mechanisms involving redox imbalance, modification of inflammatory signaling (which could be attributed to the treatment-induced reduction in cell viability), and activation of apoptotic pathways, although with distinct magnitudes and molecular signatures depending on the BC subtype.

One of the most consistent findings of this study was the induction of apoptosis by both treatments (Figure 1). While MoI produced modest increases in apoptotic activity across BC cell lines, Cv elicited a markedly stronger response, increasing apoptosis approximately 3.5–3.8-fold relative to untreated controls. Notably, this effect was observed in all BC subtypes evaluated, including the highly aggressive TNBC MDA-MB-231 cells, suggesting that the pro-apoptotic activity of Cv is largely independent of hormone receptor and HER2 status. These observations are consistent with previous reports demonstrating that oregano extracts and Cv induce apoptosis in BC cells through multiple molecular mechanisms, including activation of BAX and TP53, suppression of *BCL2*, disruption of mitochondrial integrity, and inhibition of cell-cycle regulators such as cyclin D1, CDK4, and CDK6 [11,12,13,14]. Together, these alterations promote mitochondrial dysfunction and activation of the intrinsic apoptotic pathway.

Although Cv exhibited greater pro-apoptotic potency than MoI in cancer cells, it also triggered a substantial ~4-fold increase in apoptosis in non-tumoral HaCaT cells, revealing an acute, nonspecific cytotoxicity. In contrast, Mol showed a moderate effect, suggesting a potentially more favorable therapeutic effect. Therefore, although Cv may represent the primary cytotoxic constituent, the complex phytochemical composition of MoI, previously characterized, may confer complementary biological activities while reducing toxicity toward non-tumorigenic cells [3].

Inflammation is increasingly recognized as a hallmark of cancer progression, contributing to tumor growth, therapeutic resistance, angiogenesis, and immune evasion [15,16]. In this context, cytokine profiling revealed that both MoI and Cv significantly altered inflammatory signaling pathways through extracellular cytokine concentrations, particularly in BC cells.

Among the cytokines evaluated, IL-4 emerged as a relevant target. MoI significantly reduced IL-4 production, particularly in HCC-1954 cells, whereas Cv induced a marked reduction in the extracellular concentration of IL-4 in MDA-MB-231 cells (Figure 2). This finding is biologically relevant because IL-4 promotes the expression of genes involved in cell survival, proliferation, angiogenesis, and resistance to apoptosis, including *MYC*, *BCL2*, *VEGF*, and *CCND1* [15,16]. Consequently, the reduction in IL-4 may contribute to the attenuation of tumor-supportive signaling pathways and partially explain the increased apoptotic susceptibility observed following treatment.

The effects on IL-6 were highly dependent on cellular context. MDA-MB-231 cells exhibited the highest basal IL-6 production (exceeding the upper limit of detection of the assay), consistent with the well-established inflammatory phenotype of TNBC. Importantly, Cv significantly reduced IL-6 levels in this cell line, whereas MoI decreased IL-6 in MCF-7 cells. Since IL-6 promotes tumor progression by activating pathways associated with proliferation, epithelial–mesenchymal transition, invasion, and resistance to therapy, including HIF-1α and EGFR-dependent signaling, the reduction in its extracellular concentration may represent an additional mechanism contributing to the antitumor effects observed in this study [3,15,17,18].

Changes in IL-10 production were particularly noteworthy in MDA-MB-231 cells. Both treatments reduced IL-10 secretion, with MoI producing the strongest effect. Although IL-10 may exhibit antitumor properties during early stages of carcinogenesis, established tumors frequently exploit this cytokine to suppress antigen presentation and promote immune tolerance. Consequently, reduced IL-10 production may disrupt tumor-associated immunosuppressive mechanisms and increase cellular vulnerability to apoptosis [3,15,17,18]. Supporting this interpretation, silencing IL-10 in MDA-MB-231 cells has been shown to increase caspase-3 and caspase-9 expression while reducing *PI3KCA*, *AKT*, and *BCL2* signaling, ultimately enhancing apoptotic susceptibility [18].

Interestingly, despite exhibiting greater cytotoxicity, Cv did not uniformly suppress inflammatory cytokines. In some cellular models, particularly MCF-7 and HCC-1954, Cv increased IL-10 or IL-6 production. These observations suggest that changes in extracellular cytokine concentrations by Cv may reflect complex adaptive stress responses rather than a simple anti-inflammatory effect. Nevertheless, the pronounced reduction in the extracellular concentration of IL-4, IL-6, and IL-10 observed in MDA-MB-231 cells indicates that TNBC may be particularly sensitive to Cv-mediated disruption of tumor-promoting cytokine networks [19].

These findings align with previous reports demonstrating the anti-inflammatory properties of Cv through inhibition of cyclooxygenases and lipoxygenases, thereby reducing eicosanoid synthesis and potentially interfering with signaling pathways involved in proliferation, apoptosis resistance, angiogenesis, and metastasis. Furthermore, evidence of enhanced anti-inflammatory activity when Cv is combined with thymol supports the notion that the phytochemical complexity of MoI may confer biological advantages beyond the effects of isolated compounds [19].

To date, evidence regarding the effects of oregano-derived compounds on redox homeostasis in cancer cells remains limited. Hence, a major objective of this study was to determine whether the cytotoxic effects of MoI and Cv were associated with alterations in cellular antioxidant defense mechanisms.

In addition, the most striking transcriptional alterations were observed in MDA-MB-231 cells, where both treatments reduced the expression of *CAT*, *GPX1*, and *SOD2* (Figure 3). This coordinated modification suggests a profound impairment of antioxidant defenses and a consequent increase in susceptibility to oxidative damage. Because TNBC cells are characterized by elevated basal oxidative stress and metabolic demands, further disruption of antioxidant capacity may push ROS levels beyond tolerable thresholds, thereby promoting apoptosis and enhancing therapeutic vulnerability [11,20,21,22].

A study by Nanni provides important support for the role of oregano extracts as modulators of oxidative stress in tumor cells. Using an ethanolic extract of *Origanum vulgare*, the authors demonstrated a marked reduction in the viability of murine melanoma B16-F10 cells, while exerting a substantially lower effect on non-tumoral C2C12 myoblasts. Importantly, the decrease in melanoma cell viability was accompanied by a significant increase in intracellular ROS levels, indicating that disruption of redox homeostasis contributed to the observed cytotoxicity. These findings suggest that oregano-derived phytochemicals can induce oxidative stress in cancer cells, promoting cell death while exhibiting comparatively lower toxicity toward non-cancerous cells [23].

In MCF-7 cells, Cv induced marked *SOD2* overexpression accompanied by *GPX1* downregulation (Figure 3B). This imbalance may favor hydrogen peroxide accumulation, as increased superoxide dismutation is not accompanied by proportional peroxide detoxification. Excess hydrogen peroxide can trigger mitochondrial oxidative damage and apoptotic signaling, consistent with the elevated apoptosis observed in this cell line [24,25,26].

A distinct pattern was observed in HER2-positive HCC-1954 cells. Whereas MoI increased *CAT* and *SOD2* expression, suggesting activation of adaptive antioxidant responses, Cv simultaneously suppressed *CAT*, *GPX1*, and *SOD2*, indicating a broad collapse of antioxidant defenses. Because this subtype exhibits high mitochondrial activity and basal ROS production, the decrease in *GPX1* is particularly significant, as it limits hydrogen peroxide detoxification and can therefore promote the activation of JNK/p38-dependent proapoptotic cascades, as reported in various studies [26,27].

The contrasting effects of MoI and Cv on antioxidant defense mechanisms are consistent with the dual role of ROS in cancer cells, where they function both as intracellular signaling molecules that promote adaptive responses and as mediators of oxidative damage that trigger cell death when redox homeostasis is disrupted. Moderate elevations in ROS levels can activate adaptive antioxidant responses by inducing genes such as SESN1, SESN2, PRDX1, and GPX1, thereby enhancing cancer cells’ capacity to withstand oxidative stress. Conversely, when ROS production exceeds the cellular antioxidant buffering capacity, oxidative damage accumulates and triggers pro-apoptotic signaling pathways involving mediators such as p66Shc, BAX, and PUMA, ultimately leading to mitochondrial dysfunction and cell death [20,21,22].

In non-tumoral HaCaT cells, MoI induced *SOD2* upregulation together with *CAT* and *GPX1* downregulation. Such a profile may favor hydrogen peroxide accumulation and oxidative stress if peroxide-scavenging systems become insufficient. Similar relationships between *SOD2* overexpression, impaired peroxide detoxification, and oxidative damage have been reported previously. However, the comparatively modest apoptotic response observed in HaCaT cells suggests that non-cancerous cells retain greater capacity to compensate for redox disturbances than cancer cells [19,26,27,28,29].

Overall, these findings support the hypothesis that disruption of antioxidant defenses constitutes an important component of the anticancer activity of both treatments, particularly in aggressive BC subtypes.

Although gene expression analyses revealed substantial alterations in antioxidant pathways, these changes were only partially reflected at the enzymatic level (Figure 4). Catalase activity remained remarkably stable across all cell lines despite significant transcriptional modification, suggesting that post-transcriptional regulation, protein stability, or enzyme turnover may buffer short-term functional changes.

Similarly, SOD2 activity exhibited only moderate variations despite substantial changes in transcript abundance. Such discrepancies are not unexpected, as antioxidant enzymes are subject to multiple regulatory mechanisms beyond transcription, including post-translational modifications and compartment-specific regulation.

Among the enzymes evaluated, glutathione peroxidase displayed the most pronounced functional alterations. Cv significantly reduced GPX1 activity in MCF-7 cells, whereas MoI markedly increased GPX1 activity in HCC-1954 cells. These findings may reflect adaptive responses in surviving cell populations, whereby increased antioxidant activity serves as a compensatory mechanism to counteract treatment-induced oxidative stress. Conversely, loss of GPX1 activity may amplify peroxide accumulation and contribute directly to cytotoxicity.

The limited correspondence between gene expression and enzyme activity highlights the complexity of redox regulation and suggests that longer exposure periods or direct measurements of intracellular ROS would be required to fully elucidate the functional consequences of antioxidant gene modulation.

Taken together, our results demonstrate that MoI and Cv exert anticancer effects through complementary mechanisms, including induction of apoptosis, remodeling of the cytokine network, and disruption of redox homeostasis. Although Cv displayed acute cytotoxicity, MoI produced broader modification in antioxidant and inflammatory pathways, particularly in TNBC cells.

The coordinated downregulation of *CAT*, *GPX1*, and *SOD2* observed in MDA-MB-231 cells leads us to hypothesize that MoI may sensitize BC cells to therapies that induce oxidative stress, including anthracyclines, taxanes, radiotherapy, and HER2-targeted therapies. By weakening endogenous antioxidant defenses, MoI could potentially enhance the efficacy of conventional treatments while exerting moderate toxicity toward non-tumoral cells.

Therefore, the present findings support further investigation of both Cv and MoI as complementary therapeutic strategies for BC, particularly for aggressive subtypes such as TNBC, where therapeutic options remain limited. Future studies should incorporate direct ROS measurements, mitochondrial function analyses, and in vivo validation to further elucidate the molecular mechanisms underlying their anticancer activity.

Disruption of cellular redox homeostasis has emerged as a promising therapeutic strategy for BC. This concept is particularly relevant for polyphenols and flavonoids, which exhibit concentration-dependent redox behavior. While they act as antioxidants at moderate concentrations, higher concentrations, such as those present in the MoI extract, may generate pro-oxidant intermediates that, together with the elevated basal ROS levels and metal-rich intracellular environment of BC cells, further enhance oxidative stress. Consequently, oxidative damage may exceed the antioxidant capacity of tumor cells, leading to cellular injury and ultimately cell death [3,11,20,22]. To determine whether these pro-oxidant effects were accompanied by functional alterations in antioxidant defenses, we evaluated the activity of the major antioxidant enzymes following MoI and Cv treatment.

Assessment of antioxidant enzyme activity revealed that the functional consequences of MoI and Cv treatment were considerably more limited than the transcriptional changes observed in antioxidant defense genes (Figure 3 and Figure 4). Among the enzymes evaluated, only GPX1 showed marked treatment-dependent alterations, with MoI significantly increasing GPX1 activity in HCC-1954 cells, whereas Cv significantly reduced GPX1 activity in MCF-7 cells. In contrast, CAT and SOD2 activities remained largely unchanged across all cell lines despite substantial differences in gene expression.

Importantly, the combined analysis of gene expression and enzyme activity provides a more comprehensive understanding of the redox response induced by these treatments. While transcriptional analyses indicate that both MoI and Cv alter the antioxidant defense network, particularly by modulating CAT, GPX1, and SOD2, the enzymatic data suggest that these molecular changes do not uniformly result in immediate functional impairment. Instead, GPX1 appears to be the antioxidant enzyme most responsive to treatment, highlighting peroxide detoxification as a potentially important target of oregano-derived phytochemicals. This observation is biologically relevant because disruption of peroxide metabolism may amplify oxidative stress in BC cells, thereby contributing to the enhanced apoptotic response observed, particularly following Cv treatment.

Despite the findings described herein, it is important to acknowledge certain methodological limitations of this study. First, HaCaT keratinocytes were used as a non-tumoral control. While these represent a useful non-malignant human cell model, they are not of normal mammary epithelial origin. Therefore, a definitive assessment of selectivity will require future studies using non-transformed mammary epithelial cell lines, such as MCF-10A, in comparable concentration-response experiments. Second, regarding immunological measurements, cytokine concentrations in the supernatants were not normalized to total protein content or viable cell count at the time of sampling. Consequently, the decrease in certain cytokines observed under IC_50_ conditions may be secondary to the treatment-induced reduction in cell viability, and these results should be interpreted with caution. Lastly, IL-6 levels following treatment in MDA-MB-231 cells exceeded the upper limit of detection of the assay (>500 pg/mL). Although further dilutions could not be performed to determine the exact concentration, surpassing this threshold provides robust qualitative evidence of a biologically significant hyperproduction of this cytokine induced by the treatment.

Overall, these findings demonstrate that the effects of MoI and Cv on cellular redox homeostasis involve coordinated yet only partially overlapping transcriptional and functional alterations in antioxidant defenses. Integrating both levels of regulation strengthens the evidence that disruption of oxidative stress represents an important component of the anticancer activity of these treatments and supports further investigation of oregano-derived phytochemicals as adjuvant agents targeting redox vulnerability, particularly in aggressive BC subtypes.

## 5. Conclusions

MoI and Cv emerge as promising alternative therapeutic candidates for BC. Notably, MoI led to modifications in the extracellular concentration of key cytokines (IL-4, IL-6, and IL-10); however, these regulatory effects were cell line-dependent, reflecting phenotypic heterogeneity across the tested BC models. This reduction suggests that it could interfere with biological processes crucial for cancer progression and therapeutic resistance, such as angiogenesis, proliferation, and immune evasion. Under their respective experimental conditions, both MoI and Cv exhibited distinct in vitro profiles. Specifically, treatment with MoI was characterized by a reduction in both the extracellular levels of the mentioned cytokines and the enzymatic antioxidant defense mechanisms. Additionally, in the non-tumoral HaCaT cell model, MoI demonstrated a specific cytotoxic profile at the evaluated concentrations, highlighting its distinct biological activity. Conversely, Cv displayed different anticancer properties, notably leading to an increase in the extracellular concentrations of these cytokines, a response with potentially context-dependent implications in the tumor microenvironment. Both complex natural extracts and isolated phytochemicals possess unique activities that warrant further exploration in poor-prognosis cancers, such as TNBC. Nevertheless, establishing definitive claims regarding their relative potency, safety, or selectivity requires comprehensive, equipotent concentration-response assays. Further research is needed to fully elucidate their mechanisms of action, their interactions within the tumor microenvironment, and their potential to complement conventional therapies.

## Figures and Tables

**Figure 1:**
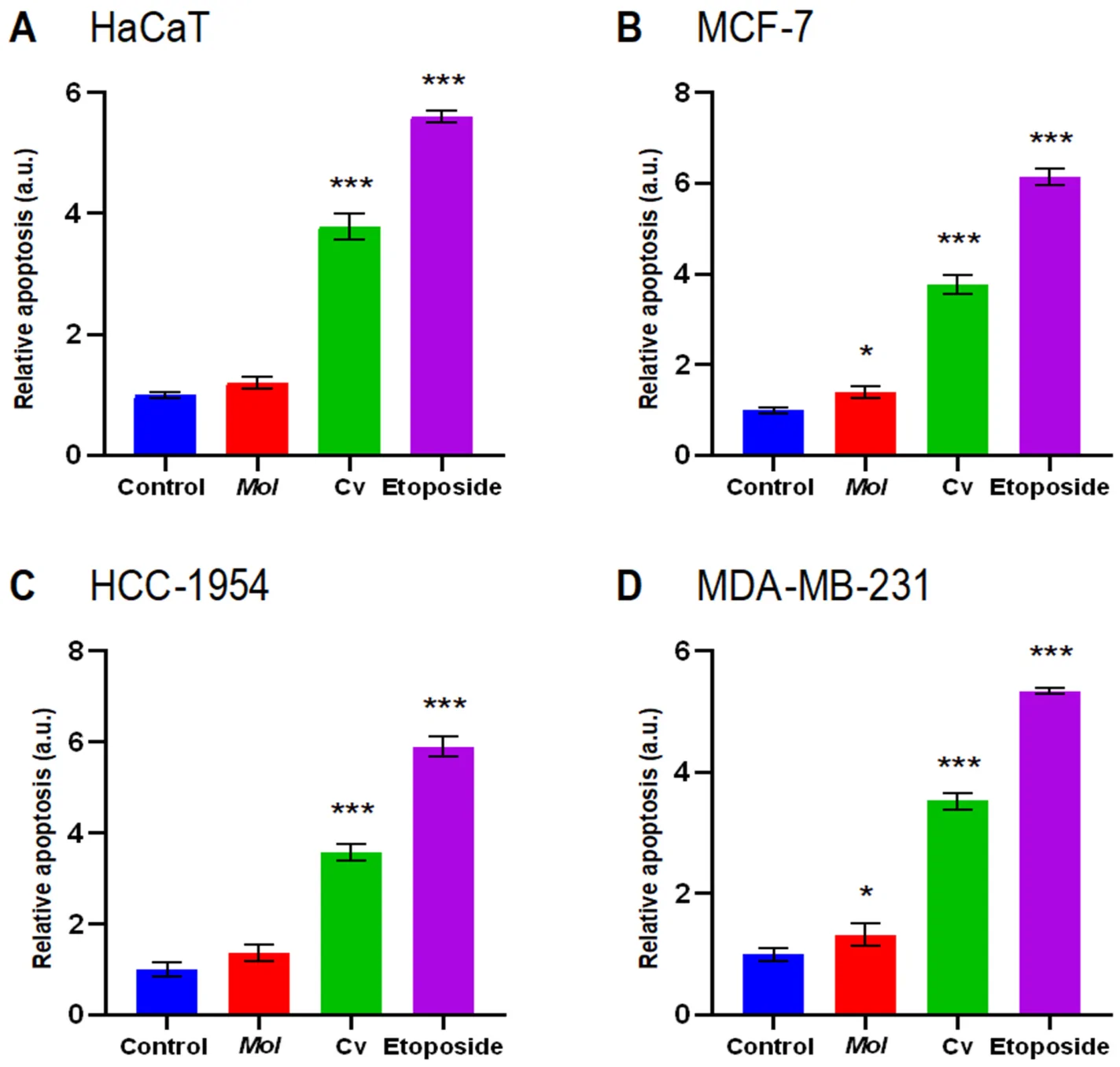
Apoptotic effect of MoI and Cv in arbitrary units (a.u.). The results were evaluated by measuring nucleosome levels in the supernatants of the following cell lines: (**A**) HaCaT, (**B**) MCF-7, (**C**) HCC-1954, and (**D**) MDA-MB-231. Each graph presents 4 experimental conditions: control; cells in the corresponding culture medium; MoI; cells treated with MoI for 48 h; Cv; cells treated with Cv for 24 h; Etoposide; cells treated with etoposide (0.18 mg/mL = 305.8 µM). Results are expressed as mean ± standard deviation; experiments were performed in triplicate at separate time points. Analysis of variance between groups was performed using ANOVA and Dunnett’s post-hoc test (* = *p* < 0.05, *** = *p* < 0.001).

**Figure 2:**
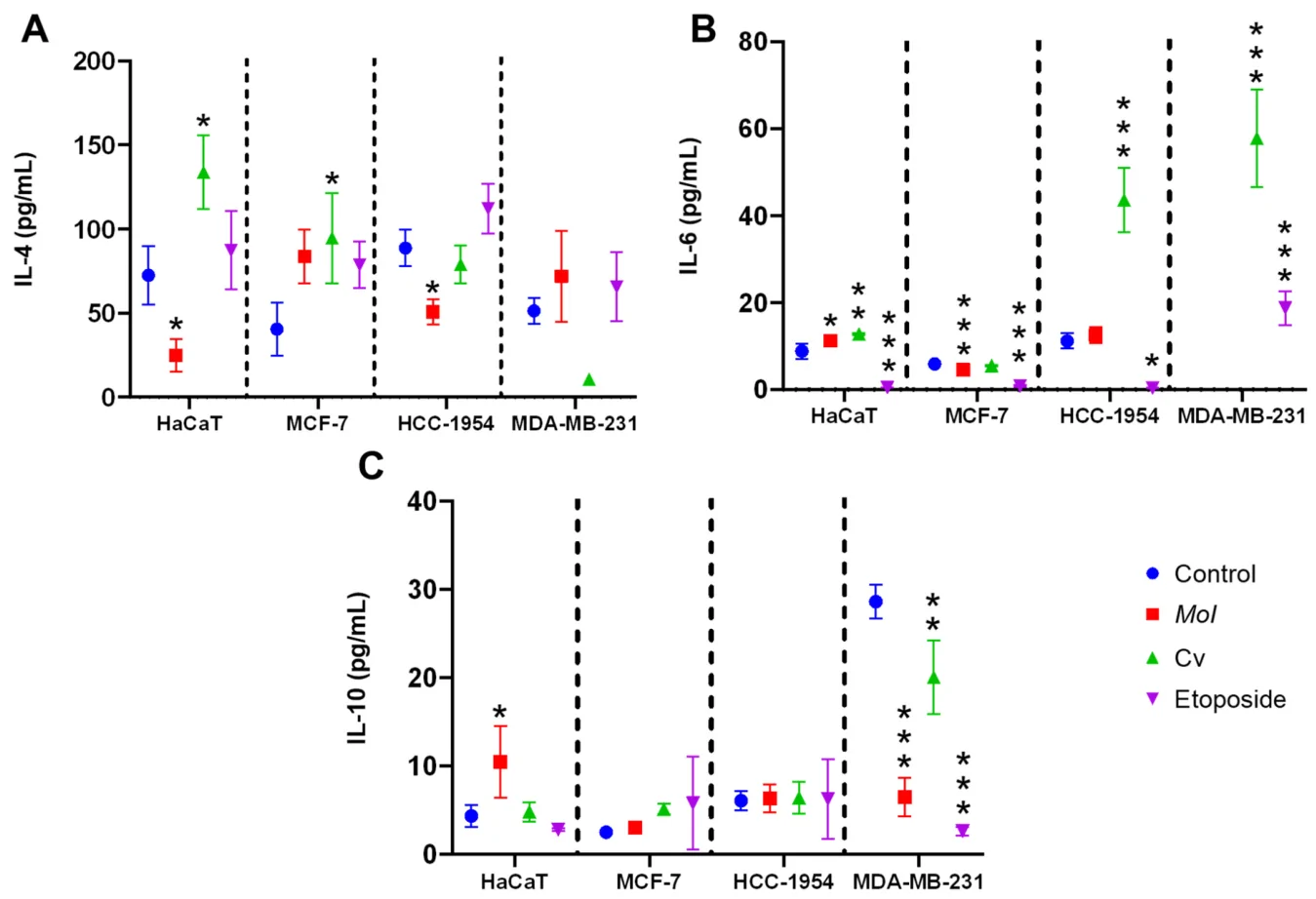
Cytokine concentration, IL-4. IL-6 and IL-10 of MoI and Cv in pg/mL. The results were evaluated by measuring the levels of (**A**) IL-4, (**B**) IL-6, and (**C**) IL-10 in the cell line supernatants. Each graph presents 3 experimental conditions: control; cells in the corresponding culture medium; MoI; cells treated with MoI for 48 h; Cv; cells treated with Cv for 24 h; Etoposide; cells treated with etoposide (0.18 mg/mL = 305.8 µM) 48 h to Mol treatment and 24 h to Cv. Results are expressed as mean ± standard deviation; experiments were performed in triplicate at separate time points. Analysis of variance between groups was performed using ANOVA and Dunnett’s post-hoc test (* = *p* < 0.05, ** = *p* < 0.01 *** = *p* < 0.001).

**Figure 3:**
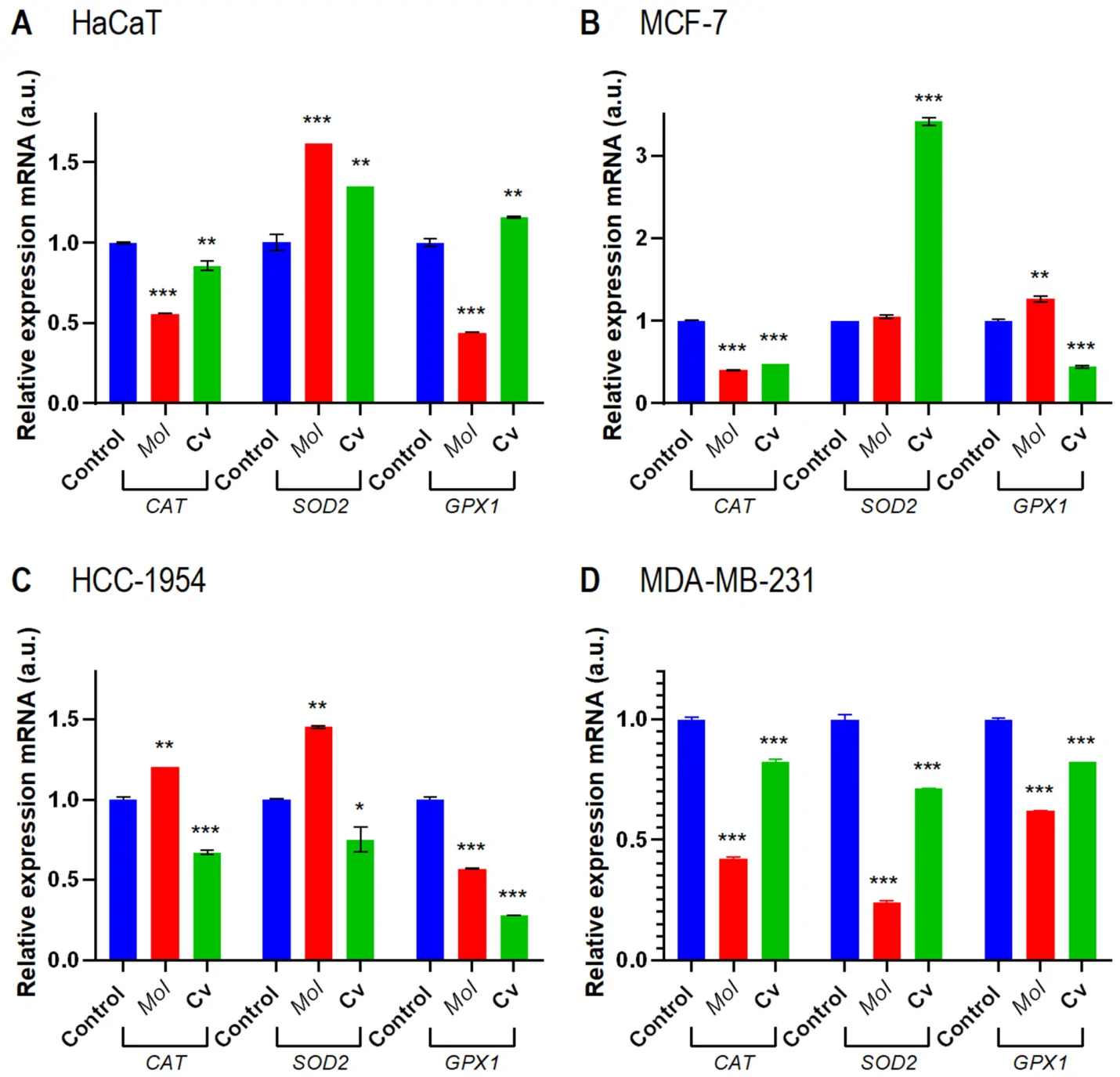
Relative expression of the *CAT*, *SOD2*, and *GPX1* genes in arbitrary units (a.u.). Results are shown grouped by cell line: (**A**) HaCaT, (**B**) MCF-7, (**C**) HCC-1954, and (**D**) MDA-MB-231; in each case, gene expression is shown under basal conditions (control) and after treatment with MoI and Cv. Relative expression was calculated using the 2^(-ΔΔCt) method, normalized with *POLR2A* as the reference gene. Bars indicate the standard deviation of the mean (±SD) corresponding to relative expression units. Analysis of variance between groups was performed using ANOVA, followed by Dunnett’s post hoc test (* = *p* < 0.05, ** = *p* < 0.01, *** = *p* < 0.001).

**Figure 4:**
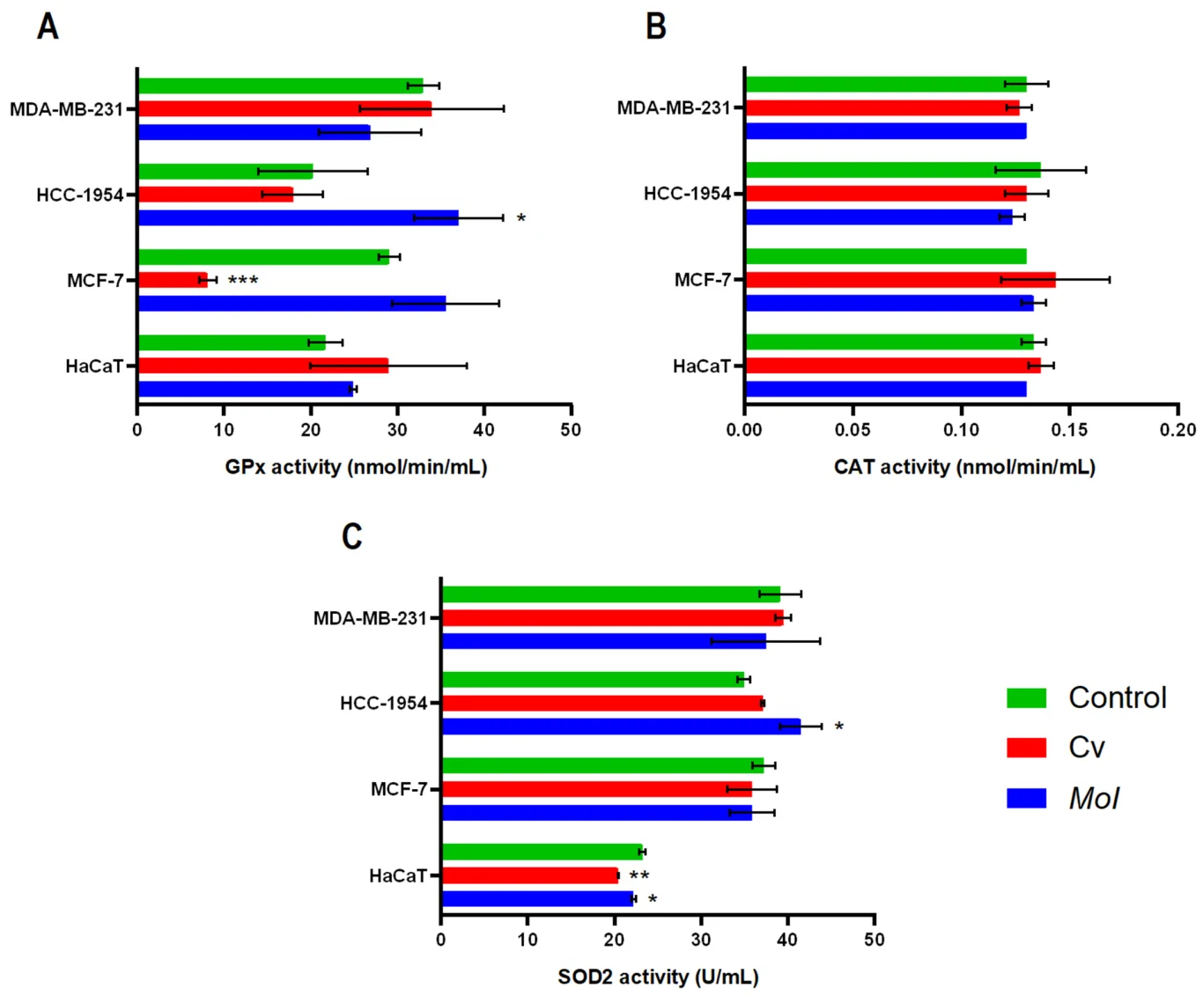
Antioxidant enzyme activities in cell lines treated with MoI and Cv. (**A**) Glutathione peroxidase, (**B**) Catalase, and (**C**) Superoxide dismutase 2. The results were determined by isolating the enzyme following treatment with the IC_50_ concentration of MoI and Cv for the corresponding time periods. Results are expressed as mean ± standard deviation; experiments were performed in triplicate at separate time points. Analysis of variance between groups was performed using ANOVA, with the Dunnett test as the post hoc test (* = *p* < 0.05, ** = *p* < 0.01, *** = *p* < 0.001).

**Figure 5:**
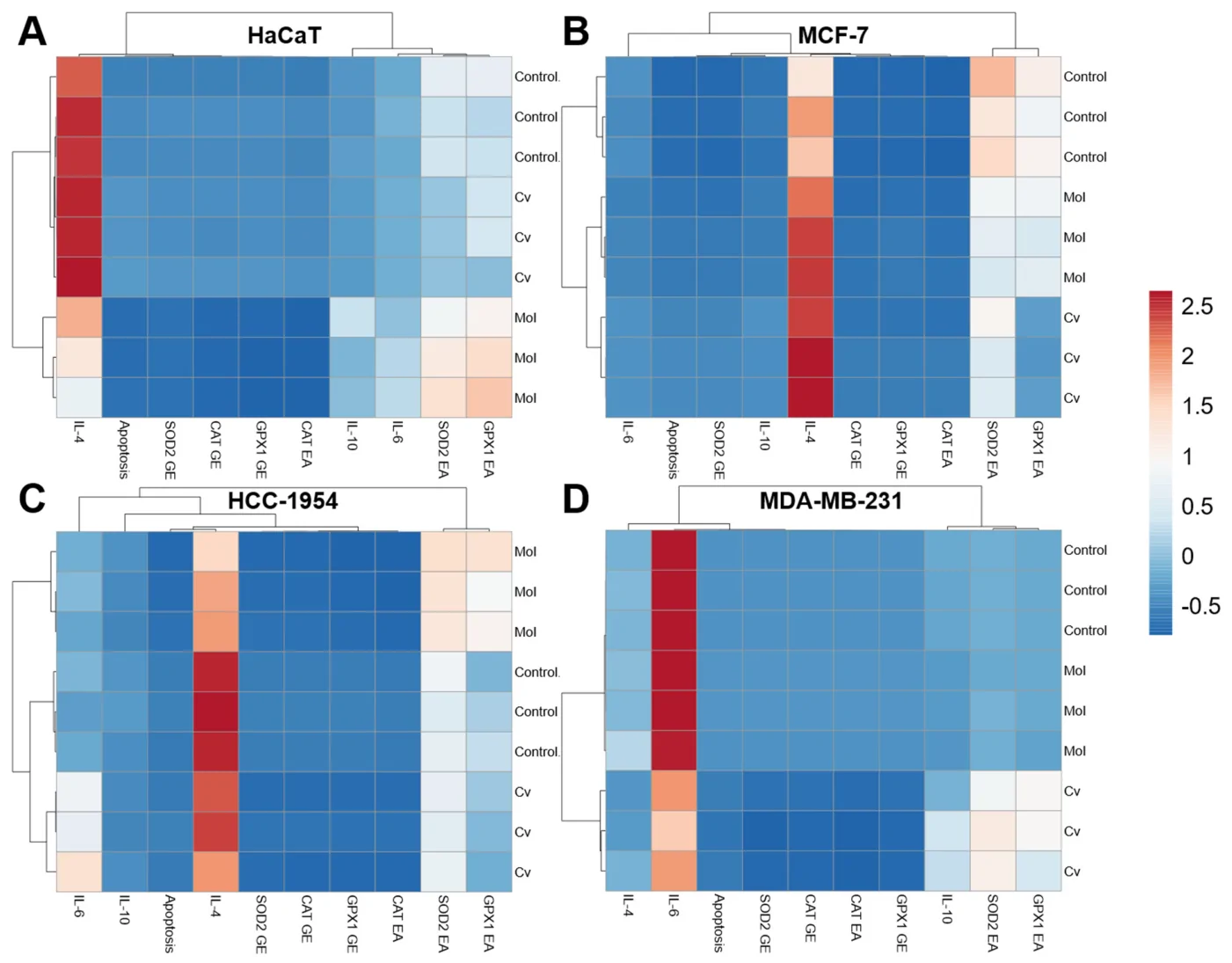
Correlation heatmap and hierarchical clustering of gene expression, antioxidant enzymatic activity (AE), cytokine production, and apoptosis across breast cancer cell lines and treatment conditions. The *Y*-axis shows the experimental conditions, including untreated control cells and cells exposed to MoI or Cv for each cell line. The *X*-axis represents the analyzed variables, including relative gene expression (CAT, SOD2, and GPX1 fold change [FC]), antioxidant enzyme activities (CAT AE, SOD2 AE, and GPX1 AE), cytokine concentrations (IL-4, IL-6, and IL-10), and apoptosis. (**A**): Represents the correlations for the HaCaT cell line; (**B**): Corresponds to the correlations for the MCF-7 cell line; (**C**): Presents the correlations for the HCC-1954 cell line; and (**D**): Shows the correlations for the MDA-MB-231 cell line. The color scale indicates the strength and direction of the correlations, with red denoting positive correlations and blue indicating negative correlations. Pearson’s correlation coefficients were calculated to assess the relationships among parameters across experimental conditions, while hierarchical clustering was applied to identify groups of variables and treatment conditions exhibiting similar response profiles.

## Data Availability

The original contributions presented in this study are included in the article/supplementary material. Further inquiries can be directed to the corresponding author.

## Supplementary Materials

The following supporting information can be downloaded at https://www.mdpi.com/article/10.3390/antiox15101345/s1.

## Abbreviations

The following abbreviations are used in this manuscript:

BC	Breast cancer
MoI	Mexican oregano infusion
Cv	Carvacrol
TKR	Tyrosine kinase receptor
DMSO	Dimethyl sulfoxide
DMEM	Dulbecco’s Modified Eagle Medium
RPMI	Roswell Park Memorial Institute
PBS	Phosphate-buffered saline
FBS	Fetal bovine serum
IBUG	Instituto de Botánica de la Universidad de Guadalajara
TMB	Tetramethylbenzidine
GPx	Glutathione peroxidase
GSH	Glutathione
GSSG	Glutathione disulfide
CAT	Catalase
H_2_O_2_	Hydrogen peroxide
KIO_4_	Potassium periodate
SOD2	Superoxide dismutase 2
O_2_^.-^	Superoxide anion radicals
SD	Standard deviation
A.u.	Arbitrary units
