# Supplementary material for: Mexican Oregano Infusion and Carvacrol Modify Cytokine Concentration, Induce Apoptosis, and Regulate Antioxidant Enzyme Expression and Function in Breast Cancer Cell Lines: Implications for Bioactive Molecule-Based Therapeutics
Source: Antioxidants (Basel). 2026 Oct 17;15(10):1345. doi: 10.3390/antiox15101345 (PMC13701643; doi:10.3390/antiox15101345)
Supplement: Supplementary file 1 [file antioxidants-15-01345-s001.zip › antioxidants-4590971-supplementary.pdf]

Nutritional, phytochemical, and antioxidant characterization of MoI.

Below is the characterization of the plant and the extract, previously published in the journal Nutrients [1].

Table S1. Bromatological analysis of the *L. graveolens*

Nutritional content	Per 100g
Energy content	363.4209.6 Kcal (1539.2876.9 KJ)
Moisture	7.8%
Ash	6.6%
Protein	6.8g
Total fat	4.2g
Available carbohydrates	36g
Dietary fiber	38.6g

Table S2. Total phenolic and flavonoids content and antioxidant capacity (DPPH, ABTS and FRAP) of MoI

ABTS	DPPH	Total phenolics	Total flavonoids	FRAP
42.837 ± 0.175 mMEQ Trolox/ g	66.303 ± 0.228 mMEQ Trolox/ g	27.765 ± 1.095 mgGAE/g	22.343 ± 0.096 mg Eq quercetin/g	109.85 ± 0.51 mMEQ FeSO4/ g

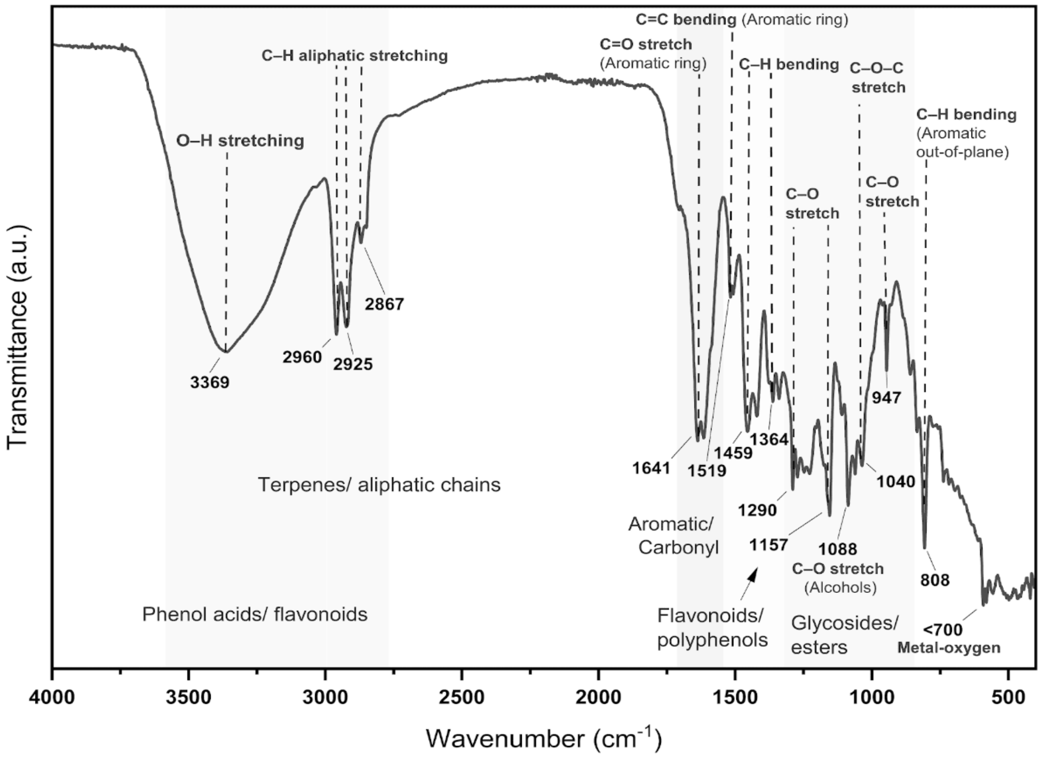

Figure S1. FTIR spectrum of Lippia graveolens infusion showing bands for phenols (~3369 cm^{-1}), terpenes (~2925–2960 cm^{-1}), aromatics (~1641 cm^{-1}), and glycosides (~1040 cm^{-1}), indicating the presence of bioactive compounds.

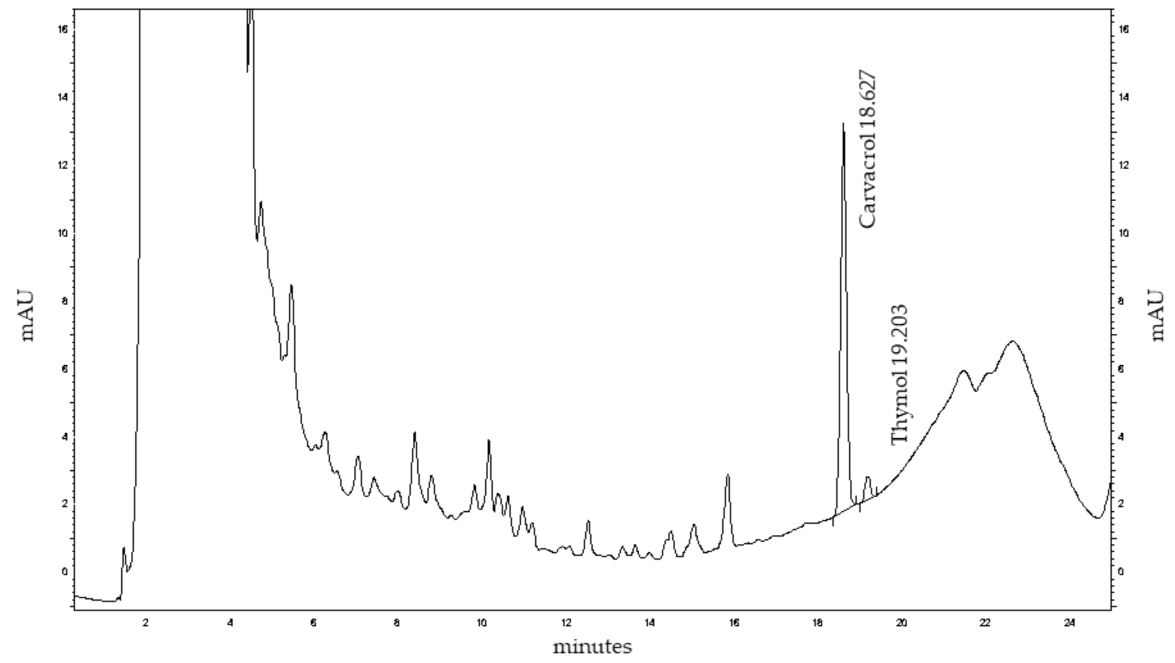

Figure S2. HPLC chromatogram of Mexican oregano (Lippia graveolens Kunth) extract at 274 nm showing major peaks for carvacrol (18.63 min) and thymol (19.20 min), along with various minor phenolic compounds.

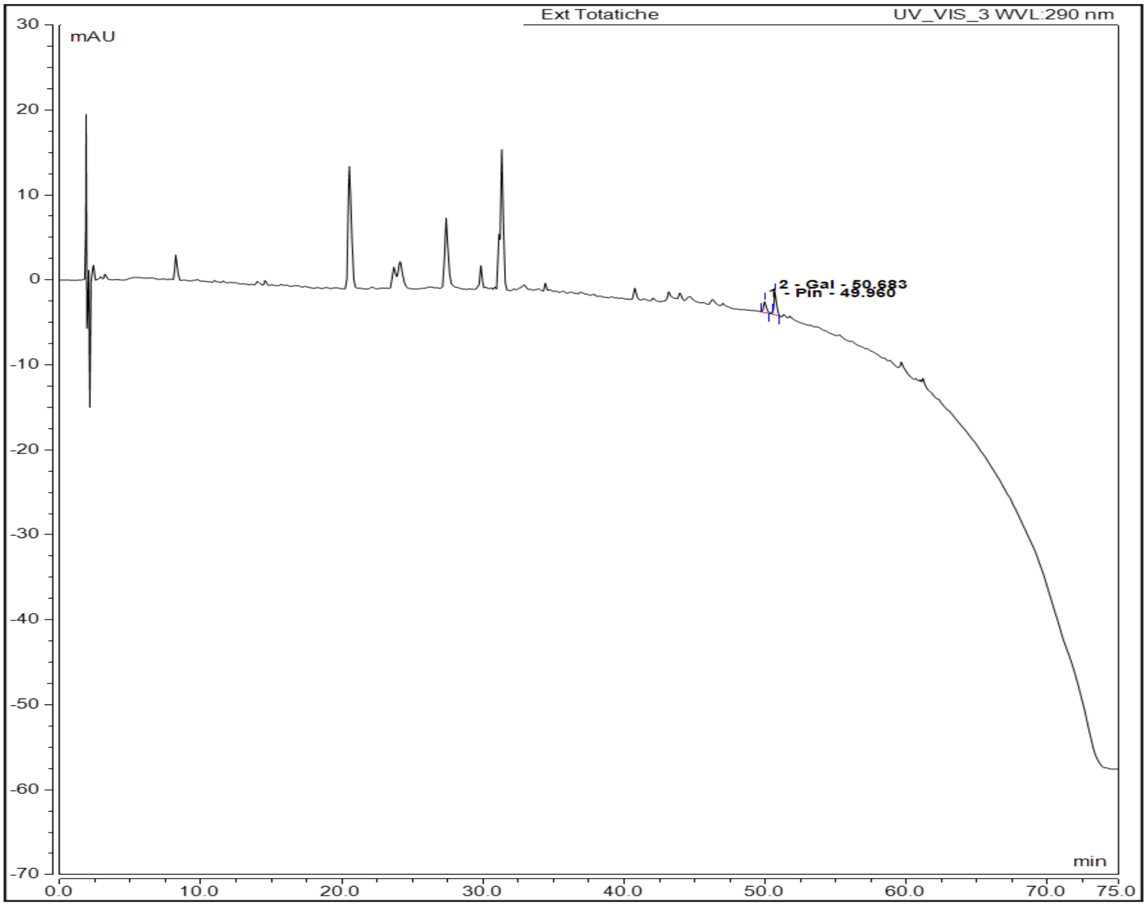

Figure S3. HPLC chromatogram of aqueous oregano leaf extract recorded at 290 nm. Pinocembrin (49.96 min) and galangin (50.68 min) were identified based on retention time and UV spectra.

1. Rojo-Ruvalcaba, B.E.; Maldonado-González, M.; Cálix-Rodríguez, G.M.; Valdés-Miramontes, E.H.; Gómez-Leyva, J.F.; García-Cobián, T.A.; Sánchez-Hernández, P.E.; Machado-Sulbaran, A.C.; López-Roa, R.I.; Balderas-León, I.; et al. Evaluation of Antioxidant-Rich Mexican Oregano (Lippia Graveolens) Infusion and Carvacrol: Impact on Metabolic Activity and Cytotoxicity in Breast Cancer Cell Lines. *Nutrients* **2025**, *17*, 3089, doi:10.3390/nu17193089.
